# Impact of Psychiatry Internship on Attitudes and Behaviors Toward Mental Illness Among Medical Students: A Cross-Sectional Study

**DOI:** 10.1192/j.eurpsy.2026.11971

**Published:** 2026-07-31

**Authors:** G. Chakchouk, N. Regaieg, Y. Trabelsi, F. Guermazi, I. Feki, J. Masmoudi, I. Baati

**Affiliations:** Department of Psychiatry A, Hedi Chaker Hospital, Sfax, Tunisia

## Abstract

**Introduction:**

Stigma toward mental illness remains a major social barrier. Exposure to psychiatric patients during training may shape medical students’ attitudes and behaviors toward people with mental illness.

**Objectives:**

To assess whether completing a psychiatry internship influences medical students’ attitudes and behaviors toward individuals with mental illness.

**Methods:**

A cross-sectional study was conducted among medical students at the Faculty of Medicine of Sfax, Tunisia, between February and October 2025. Participants completed an online questionnaire including sociodemographic data and personal experiences with mental health. Attitudes were measured using the French version of the Community Attitudes Toward Mental Illness (CAMI) scale, and behaviors using the Reported and Intended Behaviour Scale (RIBS). Data were analyzed with IBM SPSS Statistics 23.0.

**Results:**

The mean total CAMI score was 15.20 ± 3.01. Authoritarianism scores were lower among students with a psychiatry internship (13.77 ± 3.24) compared to those without (15.33 ± 2.87). Benevolence scores were high across all participants (27.50 ± 5.25 with internship and 27.61 ± 4.97 without).Social Restrictiveness was moderate (19.38 ± 4.64 with internship and 19.67 ± 5.05 without).Community Ideology was 16.73 ± 2.86 in students with a psychiatry internship ( and 16.11 ± 2.63 in those without)

The mean RIBS score was 13.41 ± 3.06, reflecting generally positive behavioral intentions. Students with psychiatry internships reported more favorable behavioral patterns (mean = 13.63 ± 3.11) than those without such experience (mean = 12.28 ± 2.76), suggesting that direct clinical contact may encourage openness and reduce avoidance behaviors.Lower Authoritarianism (p < 0.001) and Social Restrictiveness (p < 0.001) were significantly correlated with more positive RIBS scores, indicating that less stigmatizing attitudes translate into more inclusive behavioral intentions. Higher Community Ideology was also positively correlated with RIBS (p< 0.001).The Benevolence dimension showed no meaningful relationship with RIBS (p=0.217).

**Conclusions:**

Medical students demonstrated moderately positive attitudes and behaviors toward individuals with mental illness. Psychiatry internships were associated with lower stigma and more favorable behavioral intentions. Early and continuous clinical exposure in psychiatry may enhance empathy, reduce prejudice, and promote patient-centered mental healthcare among future physicians.

**Disclosure of Interest:**

None Declared

